# Detection Accuracy of Soccer Players in Aerial Images Captured from Several Viewpoints

**DOI:** 10.3390/jfmk4010009

**Published:** 2019-01-21

**Authors:** Takuro Oki, Ryusuke Miyamoto, Hiroyuki Yomo, Shinsuke Hara

**Affiliations:** 1 Department of Computer Science, Graduate School of Science and Technology, Meiji University, 1-1-1 Higashimita, Tama-ku, Kawasaki-shi 214-8517, Japan; 2 Department of Computer Science, School of Science and Technology, Meiji University, 1-1-1 Higashimita, Tama-ku, Kawasaki-shi 214-8517, Japan; 3 Department of Electrical and Electronic Engineering, Faculty of Engineering Science, Kansai University, 3-3-35 Yamate-cho, Suita-shi 564-8680, Japan; 4 Graduate School of Engineering, Osaka City University, 3-3-138 Sugimoto Sumiyoshi-ku, Osaka-shi 558-8585, Japan

**Keywords:** player detection, aerial images, informed-filters

## Abstract

In the fields of professional and amateur sports, players’ health, physical and physiological conditions during exercise should be properly monitored and managed. The authors of this paper previously proposed a real-time vital-sign monitoring system for players using a wireless multi-hop sensor network that transmits their vital data. However, existing routing schemes based on the received signal strength indicator or global positioning system do not work well, because of the high speeds and the density of sensor nodes attached to players. To solve this problem, we proposed a novel scheme, image-assisted routing (IAR), which estimates the locations of sensor nodes using images captured from cameras mounted on unmanned aerial vehicles. However, it is not clear where the best viewpoints are for aerial player detection. In this study, the authors investigated detection accuracy from several viewpoints using an aerial-image dataset generated with computer graphics. Experimental results show that the detection accuracy was best when the viewpoints were slightly distant from just above the center of the field. In the best case, the detection accuracy was very good: 0.005524 miss rate at 0.01 false positive-per-image. These results are informative for player detection using aerial images and can facilitate to realize IAR.

## 1. Introduction

A real-time vital monitoring system using wearable sensors with a wireless communication function attached to players is being developed to enhance the efficiency of training and to manage player conditions during exercise [1]. In this system, vitals (e.g., body temperature, heart rate, and oxygen consumption) are obtained using a single vital sensor attached to waist of human. Data are transmitted from all sensors and are received at a data collection server for analysis.

As is common practice, single-hop network, where all sensors are connected directly to the server, seems to be effective for the data transmission. However, this approach relies on sensors’ performance (e.g., transmission power). The requirement leads to rapid energy consumption in the sensors located at a distance from the server because they need to transmit in high power levels. Moreover, the transmitted signals are attenuated when passing through the human body or other obstruction.

Instead, our system adopts multi-hop network, which is more appropriate for large-scale networks where the server is located far from the sensors. In the network, each sensor behaves as a start or relay node, and vital data are transmitted to a server, an end node, like a bucket brigade.

This network is helpful for avoiding the rapid energy consumption and the wave-shielding effect of the human body. To construct an appropriate network, the accurate information about sensors’ locations is required. Received signal-strength (RSSI)- or global positioning system (GPS)-based schemes are widely used for this purpose. However, RSSI value is easily affected by some interference such as multipath fading and any obstacles, and GPS cannot accurately estimate the positions in real-time. Therefore, these schemes cannot be applied to our system, because the system should be available even when both the moving speed and the density of vital sensor nodes could be very high in many kinds of sporting events (e.g., soccer, basketball, rugby, and hockey).

To solve this problem, therefore, we proposed a novel scheme, image-assisted routing (IAR), in a previous study [2]. In this scheme, the locations of sensor nodes are estimated by finding the locations of players wearing sensors using sports-scene images and image processing techniques. To obtain images necessary for IAR, several cameras, mounted on unmanned aerial vehicles (UAV) or fixed tripods, are placed around the playing field. Additionally, whereas IAR can be expected to be applied to many sports, this paper focuses on and discusses field sports (i.e., soccer).

Tracab [3] is one of the practical systems that estimates players’ position and visualizes the statistics of their performance during field games. However, the current positioning scheme used for the system are very large and expensive because it employs patented image processing techniques developed for military purposes. Therefore, this system can be equipped only at large stadiums, although it is strongly required by club teams. To provide more reasonable system, we need to achieve an accurate localization that does not require special technologies and huge computational resources.

To achieve the desired localization, players’ locations must be accurately estimated in sports movies using visual-object detection technique. This is one of the most challenging tasks in the field of computer vision. To solve this task, several kinds of schemes have been proposed [4,5,6,7,8]. Recently, deep convolutional neural networks (CNN) have shown good accuracy for image classification. Thus, they have begun to show good accuracy for visual-object detection. The most popular type is the region-convolutional neural network (R-CNN). Several derivatives (e.g., Fast R-CNN [9] and Faster R-CNN [10]) have been proposed to improve computational speeds and detection accuracy.

However, it is shown that the detection accuracy with informed-filters [8] using only color features is better than these schemes for player detection in soccer scenes [2,11]. However, informed-filters is just a traditional object detection method built on handcrafted features. The computation speed of the scheme based on informed-filters can be improved by parallel processing with a graphical processing unit without degrading detection accuracy [12]. Therefore, informed-filters is more suitable for embedded systems for IAR than schemes based on CNN in terms of detection accuracy and computational resources for real-time processing.

The accuracy of the player detection scheme based on informed-filters using only color features was evaluated using top-down view images generated from a computer graphics (CG)-based dataset where the locations of players were determined from an actual soccer scene. However, in previous studies [13,14,15], the relationship between camera locations and detection accuracy have not been sufficiently investigated. However, it seems important for player detection using aerial images.

The goal of this paper is to extend our previous research and find the optimal camera locations for player detection in aerial images obtained from UAVs. To achieve this goal, multi-viewpoints datasets were created. They comprise frames captured from cameras placed in a three-dimensional virtual soccer field. Then, we constructed classifiers for player detection, and evaluated detection accuracy according to several viewpoints, using these datasets. Experimental criteria and results are shown below, and we believe these results are informative for player detection using aerial images and can facilitate to realize IAR.

**Detection accuracy according to multiple viewpoints.** We evaluated the detection accuracy for several viewpoints and find good one for player detection. Detection accuracy was best where the viewpoints were slightly distant from just above the center of the field. However, the detection accuracy was worse when the height of viewpoints were lower because of increasing occlusions.

**Comparison with another object detection method.** We compared our scheme, using informed-filters, with YOLOv3 [16], a state-of-the-art scheme based on deep-learning in object detection. Our scheme using outperformed the accuracy of this scheme. In particular, our scheme achieved very low FPPI while YOLOv3 quite large.

**Detection accuracy using multiple detectors specialized for only some viewpoints.** A detector constructed with training samples extracted from all viewpoints was used for the previous evaluation. However, in this evaluation, viewpoints were divided into groups, and multiple detectors specialized for only some viewpoints were constructed. We evaluated detection accuracy using the same evaluation criteria as the previous experiment, and detection became better and more stable.

## 2. Datasets Used in Our Evaluation

This section details datasets used in our evaluation. Whereas we wished to create datasets from multiple viewpoints, it would cost a lot to prepare one for a real image. Therefore, by the three-dimensional computer graphics (3DCG) technology, we constructed novel datasets from an actual soccer game and used it for training and evaluation.

### 2.1. True Locations Obtained from Actual Motion of Players

**Figure 1:**
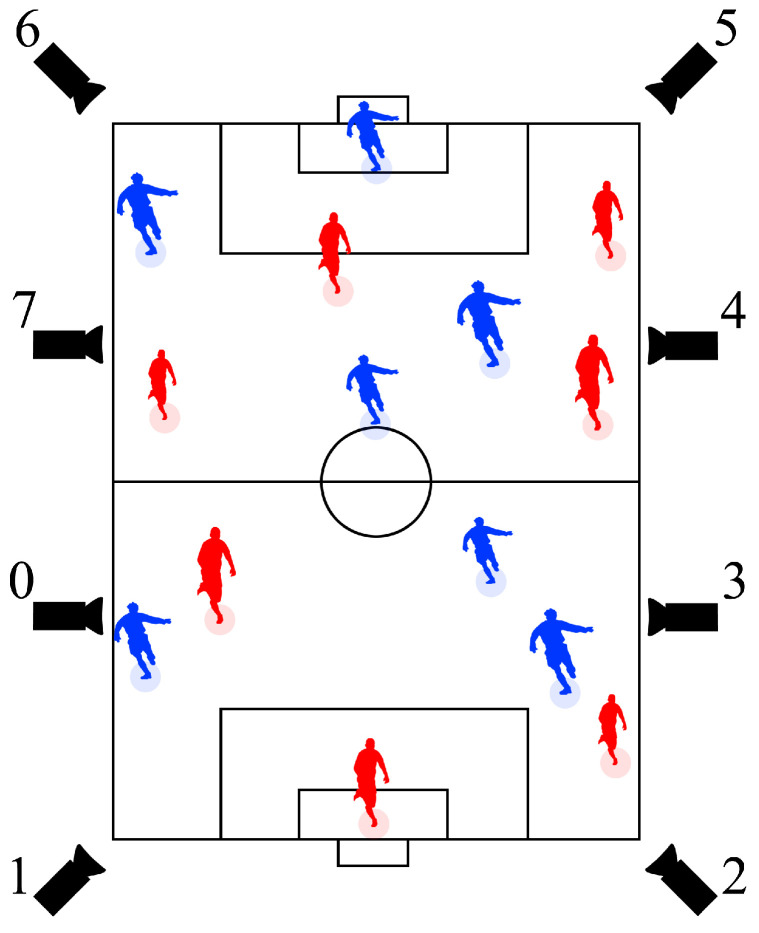
Camera locations.

**Figure 2:**
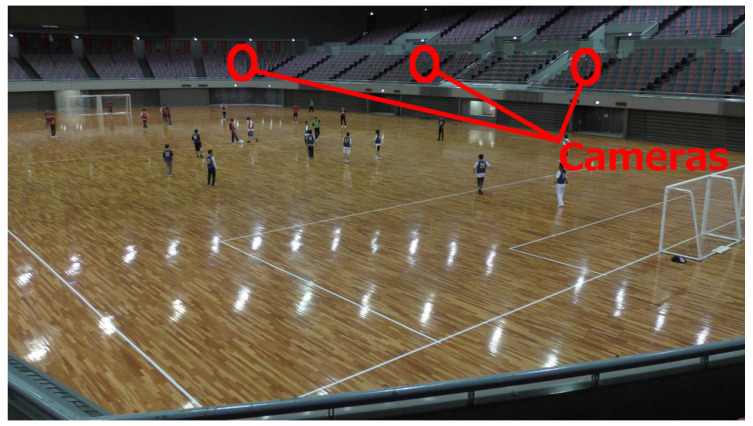
Example of captured image.

**Figure 3:**
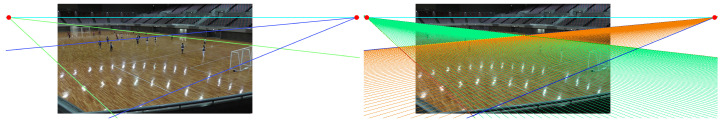
The soccer field in the captured image can be equally divided by lines drawn from two vanishing points. The vanishing points were obtained by first considering the field is rectangular in shape. By combining this vanishing point with the perspective technique, it was possible to divide the field into an arbitrary number of divisions. However, it was not strict.

**Figure 4:**
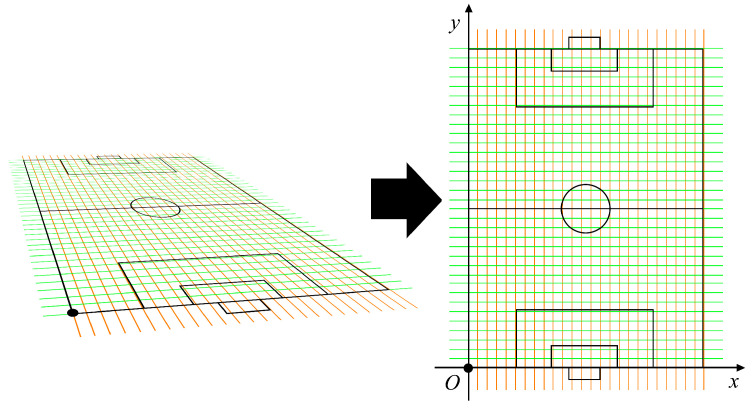
Overview of the coordinate transform. The coordinate system to the right is orthogonal, for which the black dot is set as the origin. The short side of the soccer field is set as the x-axis. The long side is set as the y-axis. Because coordinates are associated from both left and right spaces, if we know the coordinates of the player in the left space, the coordinates in the right space can be easily obtained.

The motions of players from the dataset used in our experiment are identical to those from a dataset created in our previous research [17]. The dataset was created using actual motions of players in a soccer game obtained from 9000 image sequences captured by cameras located around a soccer field (see Figure 1 and Figure 2). To determine actual locations of players in a frame, rectangles representing player locations in captured image sequences were marked manually. After marking all players in the dataset, as shown in Figure 3, the vanishing points were obtained. Then, the equal division lines of the field could be calculated from them. A two-dimensional (2D) location in the 2D image plane was determined by the center of the bottom edge of a rectangle. Then, a location on the soccer field was obtained via coordinate transform. Figure 4 shows the overview of the coordinate transform from an image plane to a soccer field.

**Figure 5:**
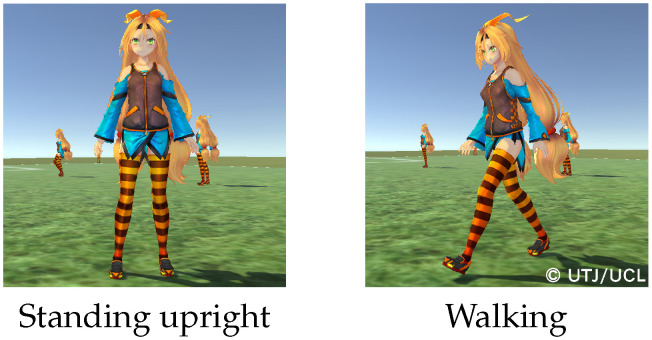
Example of a 3D model: Unity-chan.

After the creation of the ground truth about player locations, a three-dimensional (3D) virtual space representing players’ motions from the soccer game was created using Unity engine, where Unity-chan, a virtual character having the 3D model shown in Figure 5, was adopted to represent players on the soccer field. By using the virtual space, represented by the 3DCG technology, we could easily obtain images captured from arbitrary viewpoints.

### 2.2. Datasets Generated by the 3D Virtual Space for Several Viewpoints

The main purpose of this study was to evaluate detection accuracy according to several viewpoints for aerial images obtained from a camera mounted on a UAV. Therefore, several kinds of datasets were created from the 3DCG virtual space [17], changing locations and orientations of a camera in the space. Locations of players in the obtained images were generated automatically.

**Figure 6:**
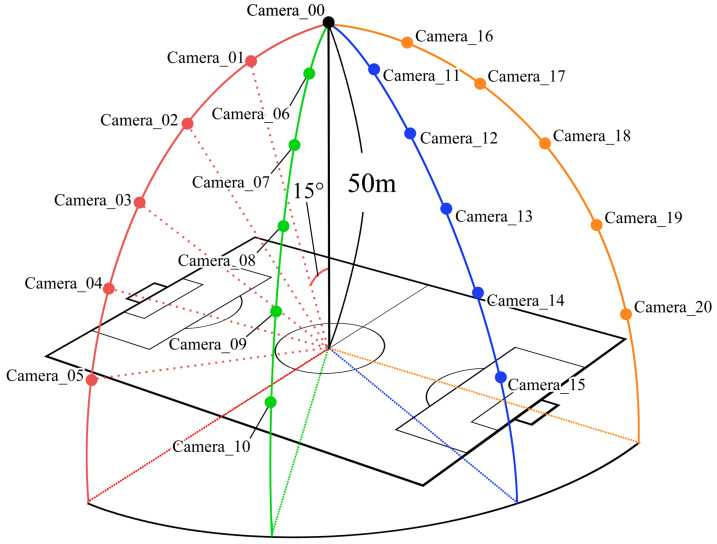
Overview of camera locations.

Figure 6 provides the viewpoints used to generate 2D images from the 3DCG virtual space. The height of the camera when located at just above the center of the soccer field was set to 50 m in the virtual space. This location was indicated as Camera_00. The camera location moved on four circumferences: red, green, blue, and orange. Here, the angle between nearest radii was set to 15°, and the camera orientation was set to the opposite radial direction.

**Figure 7:**
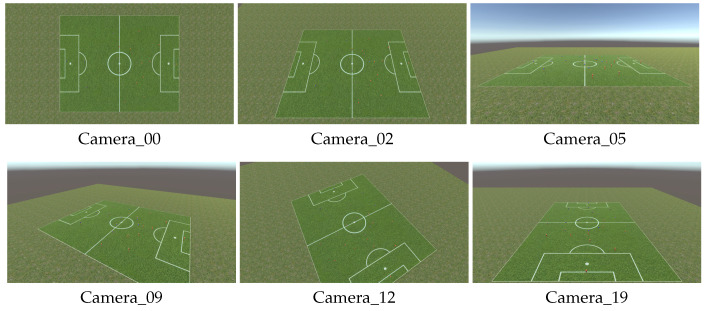
Examples of datasets.

Figure 7 shows examples of generated images as the datasets for evaluation in this study.

## 3. How to Construct a Detector Based on Informed-Filters

This section details how to construct a soccer player detector using only color features with informed-filters to evaluate the detection accuracy according to several kinds of viewpoints. The rest of this section describes training samples selection, template pool design, required for learning with informed-filters, and a final strong classifier.

### 3.1. Training Samples

**Figure 8:**
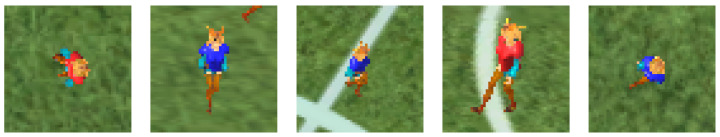
Positive samples.

**Figure 9:**
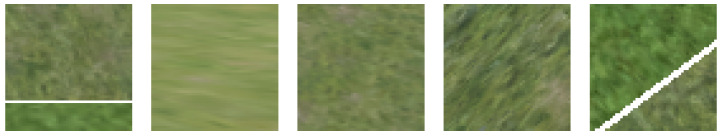
Negative samples.

Datasets used for the evaluation comprise 2D image sequences according to several viewpoints generated from the 3DCG virtual space, as shown in the previous section. To train a classifier as a detector, positive and negative samples must be extracted from the images generated from the 3DCG virtual space. Figure 8 shows examples of positive training samples, where players are located at the center of cropped sub-images. The dataset we used was generated from the 3D virtual environment using Unity. Thus, players’ locations in an image captured from a camera can be easily calculated. Negative samples were randomly cropped. They did not include players in cropped sub-images, as shown in Figure 9. The positives and negatives were extracted from 7200 frames, randomly selected from 9000 frames in the dataset. The number of both is 30,000 samples.

During generation of positive samples from image sequences using the actual location of targets obtained from the 3D virtual space, occlusions sometimes occurred when viewpoints moved drastically. Such occluded samples were not included in the training and evaluation samples, because they were not suitable for appropriate evaluation. After creating the training samples, a strong classifier was constructed using the boosting algorithm with feature extraction and templates, as described below.

### 3.2. Template Design for Informed-Filters

**Figure 10:**
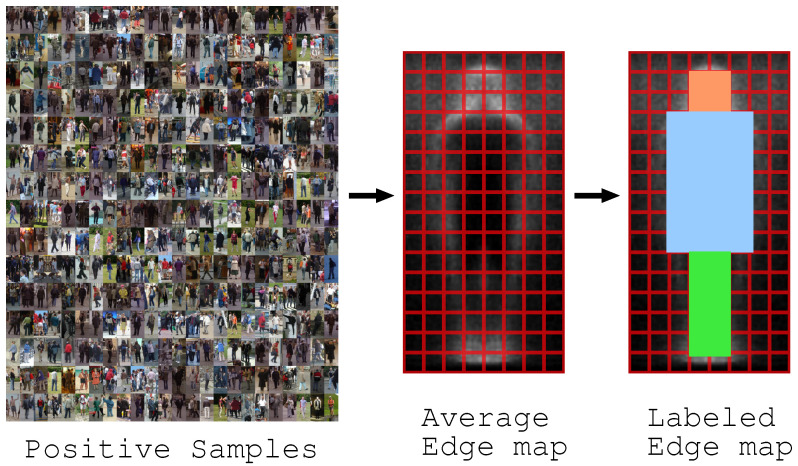
Generate edge map.

**Figure 11:**
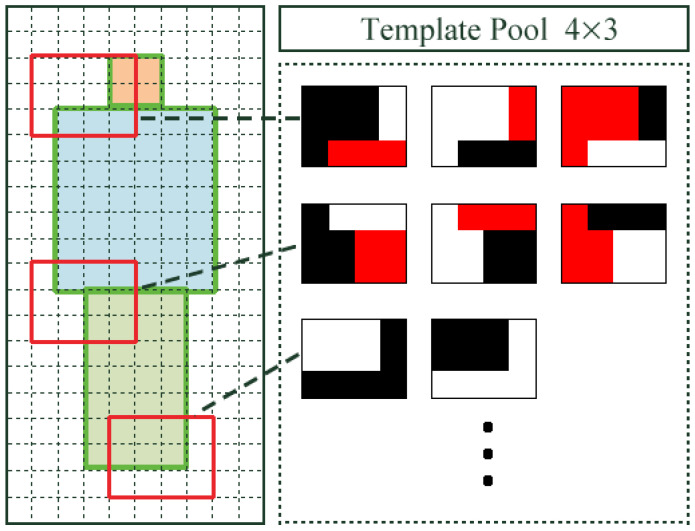
Template generation.

The training process of a detector by informed-filters is summarized in Figure 10 and Figure 11. Informed-filters [8] extracts effective features, owing to its well-designed template pool, using the statistical shape information of objects to be detected, enabling accurate human detection.

To design such template pool, the overall procedure can be summarized as follows.

1. An average edge map is computed from cropped positive samples with canny a edge detector, and is divided into cells of 1×1 pixels.
2. An average edge map is roughly classified into three parts: head, upper body, and lower body.
3. Each cell is assigned +1, 0 or −1 to distinguish which cells belong to which parts. The result of labeling is a labeled-edge map, as shown in Figure 10. The labeled-edge map represents a statistical human shape with strong patterns. Moreover, regions near the head and shoulders are uniquely human.
4. Templates are generated as a collection of cells from the labeled-edge map via an exhaustive search. The size of the template is within a range from 1×2 to 6×8 cells.

It is obvious that positive training samples were necessary for the template design described above. However, a good template pool might not be obtained if inappropriate samples were used for edge-map generation. Furthermore, the selection of positive samples for template design becomes important because the appearance of players greatly differ when viewpoints change drastically.

To avoid this problem, a template pool for informed filters was generated as follows:

1. Divide all viewpoints into five groups according to shooting orientations.
2. Generate edge maps for each group.
3. Create template pools according to generated edge maps.
4. Merge all template pools obtained in the previous procedure to generate a large template pool.

**Figure 12:**
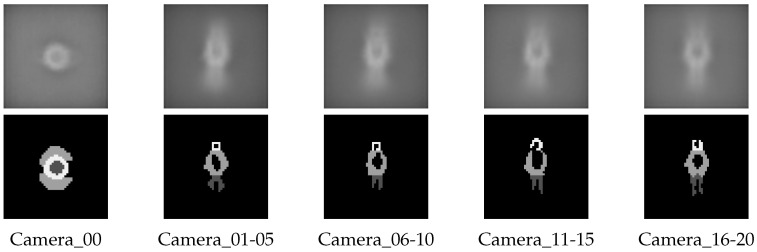
Average edge maps and labeled edge maps according to shooting orientations. Top and bottom images represent average and labeled-edge maps, respectively.

**Figure 13:**
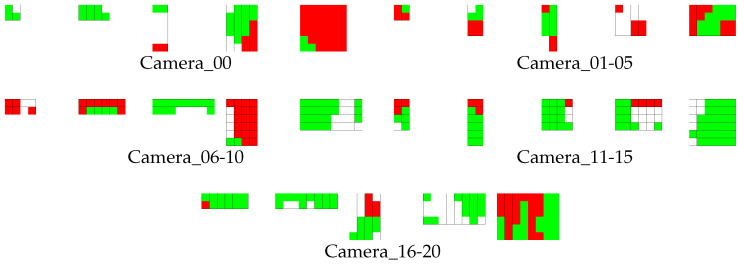
Examples of templates used to construct a classifier for players.

This procedure tries to maintain unique characteristics according to shooting orientations in the generation of a template pool. Figure 12 shows an average edge map and a labeled-edge map according to shooting orientations. Using these shape models, we obtained 36,446 templates, as shown in Figure 13.

After the generation of the large template pool, a strong classifier ws constructed. During the training process, the boosting algorithm selected effective weak classifiers from the large template pool.

## 4. Experiments to Find the Optimal Camera Viewpoints for Player Detection

We investigated a good viewpoint for player detection from a camera mounted on a UAV. We performed three experiments on the 3DCG-based dataset. First, all viewpoints were evaluated by each detection accuracy. Then, we also evaluated the accuracy with another scheme under the same conditions as first experiment, and compared them. Finally, for the further improvement, we constructed multiple detectors specialized for only some viewpoints and verified how the accuracy was affected.

In our experiments, we did not use the same frames for training and testing. For testing, each viewpoint has 1800 frames generated from the same 1800 image sequences in the 3D virtual space. To train the detector, the other 7200 frames for each viewpoint were used.

### 4.1. Detection Accuracy According to Camera Locations

This section presents the evaluation of the detection accuracy for several viewpoints to find a good one for player detection from a camera mounted on a UAV. This experiment was a simple and immediate method for our purpose.

#### 4.1.1. Detection Procedure

Detection was performed using an exhaustive search with sliding windows, which extracted huge numbers of sub-images from an input image to determine whether the extracted sub-image included a detection target or not. During this process, scaled images were generated to find targets with resolutions different from the size of the detection window. Because the size of detection targets did not change drastically in aerial images, this evaluation used only three scales: ×1.0, ×1.05, and ×1.1. The size of the stride for moving the sliding windows was one, meaning all locations of an input image were evaluated.

For the evaluation, a detected sub-window was accepted as a true positive if $Score_{overlap}$, computed by the following equation, was greater than 0.65:

(1) $$\begin{array}{c} Score_{overlap}=\frac{BB_{GT}\cap BB_{DET}}{BB_{GT}}, \end{array}$$

where $BB_{GT}$ and $BB_{DET}$ indicate a sub-window defined in ground truth and a detected sub-window, respectively.

#### 4.1.2. Experimental Results

Table 1, Table 2, Table 3, Table 4 and Table 5 show the evaluation results of detection accuracy. In these tables, miss rates at some false positives-per-image (FPPI) are shown.

These results show that very accurate detection can be achieved at all viewpoints. The best accuracy was obtained by Camera_17. The primary method of improving detection accuracy was increasing the visual cues according to the increase of the projected area of detection targets, which became the smallest at Camera_00. However, the detection accuracy became worse when the height of viewpoints was too low, because heavy occlusions may be caused at such viewpoints.

#### 4.1.3. Discussion

**Figure 14:**
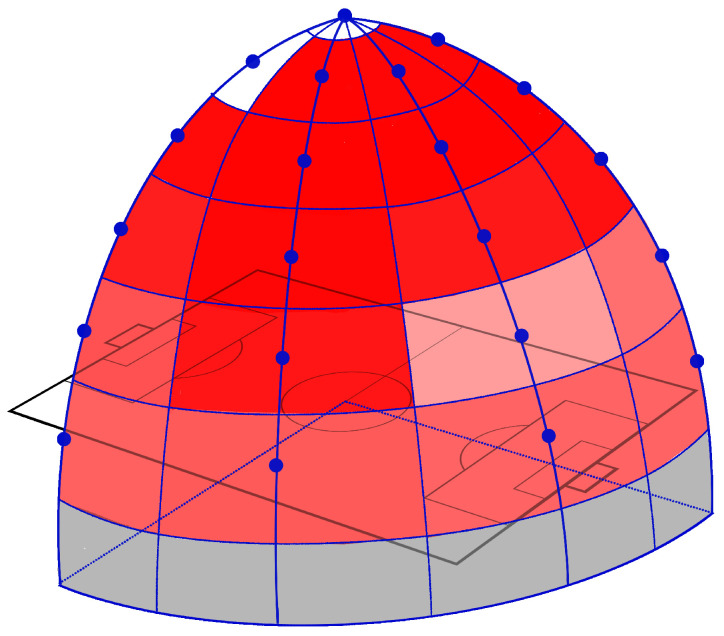
Heat map.

Figure 14 shows the heat map, representing the detection accuracy at all viewpoints, defined in Figure 6 when FPPI was 0.001. In this figure, the deep-red color indicates a lower miss rate (higher accuracy), and the miss rate becomes greater if the red components become lower. Regions colored with gray were not used in the evaluation, because detection accuracy became worse if the height of viewpoints was too low because of occlusion caused by other targets. Two regions representing Camera_00 and Camera_01 are white. The detection accuracy was quite bad in these regions. However, we found that these cameras achieved good accuracy similar to the other cameras when FPPI was 0.01 or larger (Table 1, Table 2, Table 3, Table 4 and Table 5). This fact indicates that the training of detector was incomplete, and accuracy suddenly changed between 0.001 and 0.01. To further improve the performance, collecting more training data or resampling the dataset for balancing positives and negatives can be applied to increase the stability of learning.

### 4.2. ACCURACY COMPARISON WITH YOU-LOOK-ONLY-ONCE (YOLO)

Next, we evaluated the detection accuracy by YOLO [18,19] using the same dataset, to compare the accuracy. YOLO is one of the most famous and accurate object detectors based on deep learning. In the evaluation, the latest implementation (i.e., YOLOv3 [16]) was adopted.

#### 4.2.1. Object Detection Using YOLO

Many object schemes adopt exhaustive searches based on sliding windows, where huge sub-windows of several sizes and locations are densely sampled from an input image to detect objects at all locations with several sizes. However, these traditional schemes require a classification for each sub-window. It may lead to huge computational costs, depending on resolution of input image.

YOLO is a new real-time object detection method based on regression. Instead of using the sliding windows, YOLO divides the image into S×S grid and predicts each grid with regression. A fixed number of bounding boxes are given to grid cells, and each grid cell predicts confidence scores for those boxes. The confidence scores are used to calculate how probability the grid cell contain an object and how accurate the bounding boxes is.

#### 4.2.2. How to Train YOLOv3

In this evaluation, a detector was trained for 50K iterations on the same datasets used in previous experiments. The resolution of input images was set to 416×416 and the batch size was set to 64.

#### 4.2.3. Experimental Results

Table 6, Table 7, Table 8, Table 9 and Table 10 show miss rates for several FPPIs. We found that there were mostly no value in these tables except for Table 6. The “nan” in the table indicates that FPPIs, calculated in the evaluation process, could not reach the specified FPPIs (i.e., 1.0, 0.1, 0.01, and 0.001), because there were many false positives.

**Figure 15:**
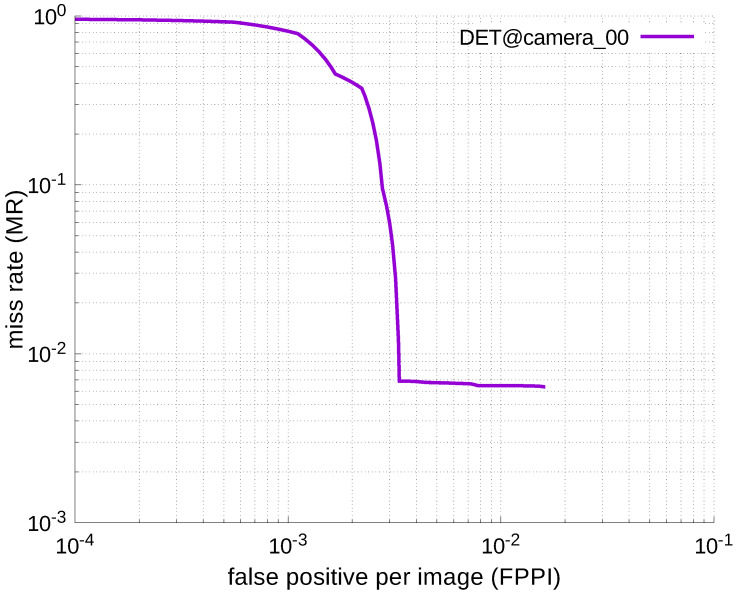
DET curves @ Camera_00, informed-filters.

**Figure 16:**
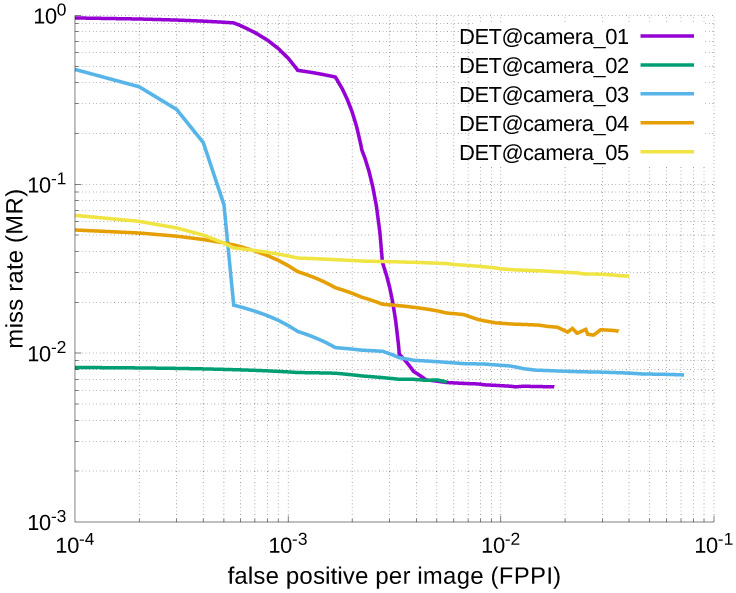
DET curves @ Cameras_01–05, informed-filters.

**Figure 17:**
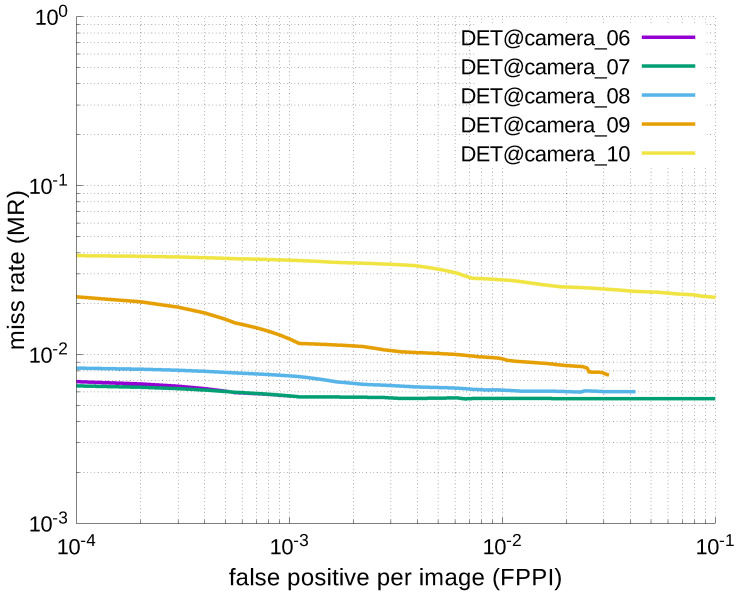
DET curves @ Cameras_06–10, informed-filters.

**Figure 18:**
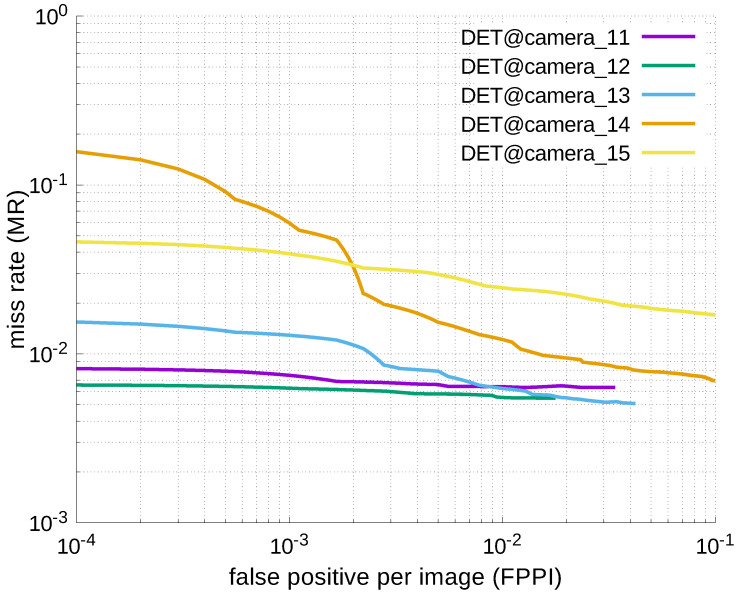
DET curves @ Cameras_11–15, informed-filters.

**Figure 19:**
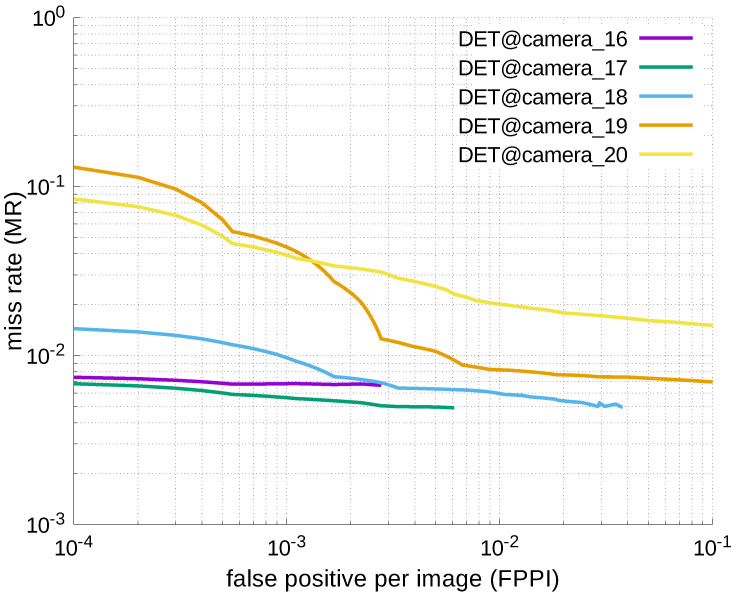
DET curves @ Cameras_16–20, informed-filters.

**Figure 20:**
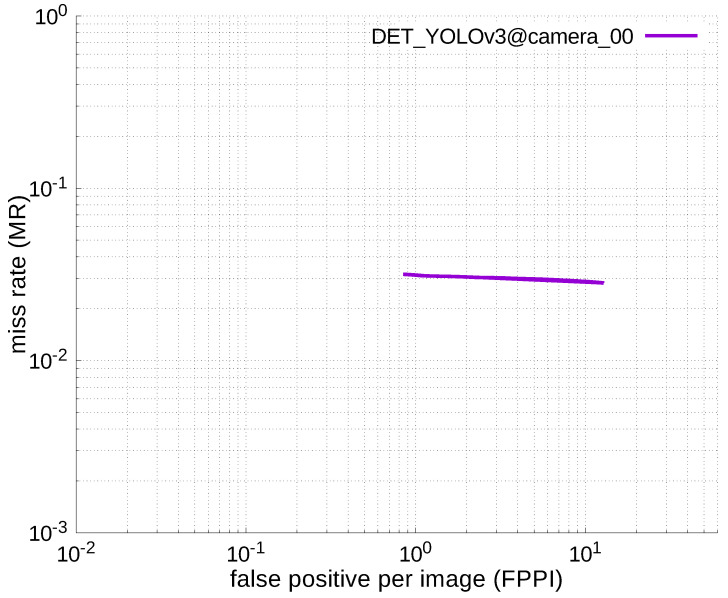
DET curve @ Camera_00, YOLOv3.

**Figure 21:**
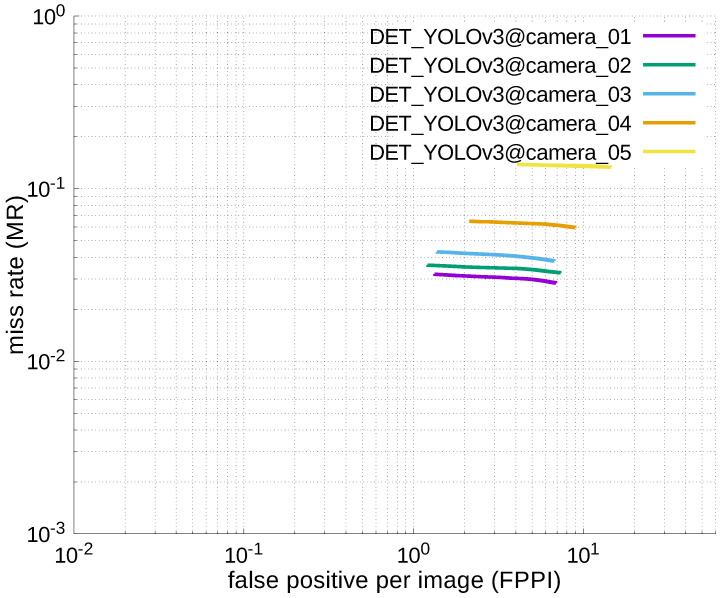
DET curves @ Cameras_01–05, YOLOv3.

**Figure 22:**
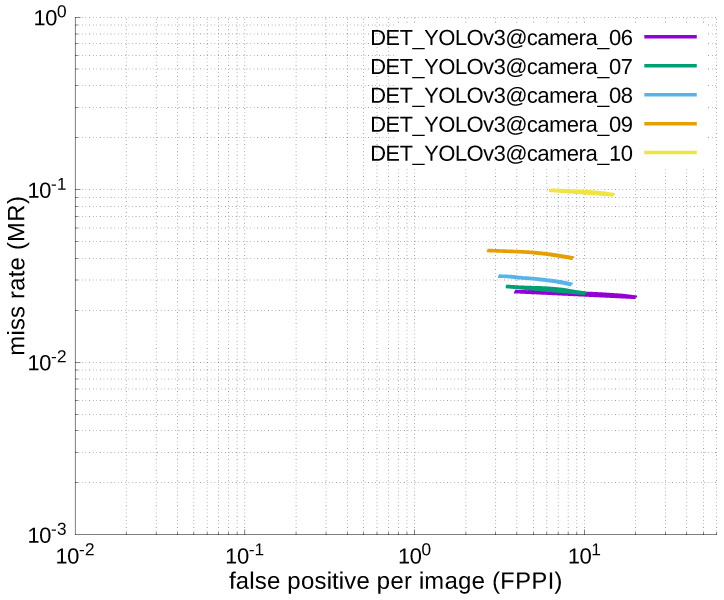
DET curves @ Cameras_06–10, YOLOv3.

**Figure 23:**
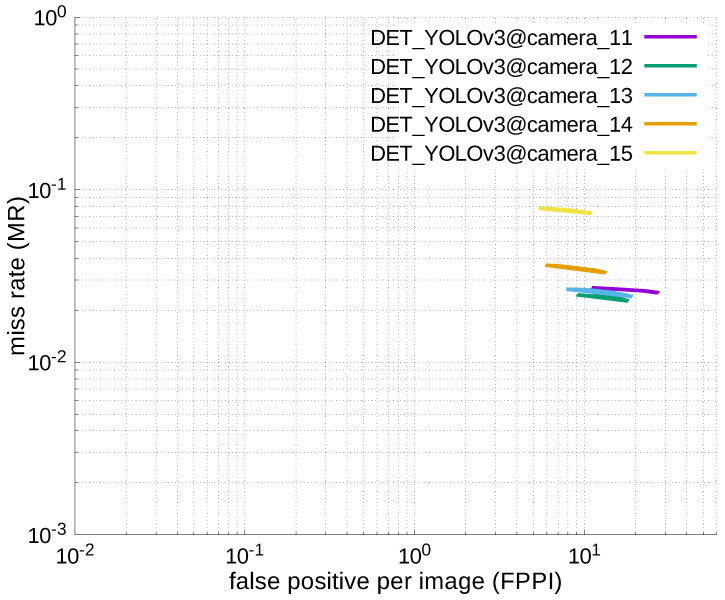
DET curves @ Cameras_11–15, YOLOv3.

**Figure 24:**
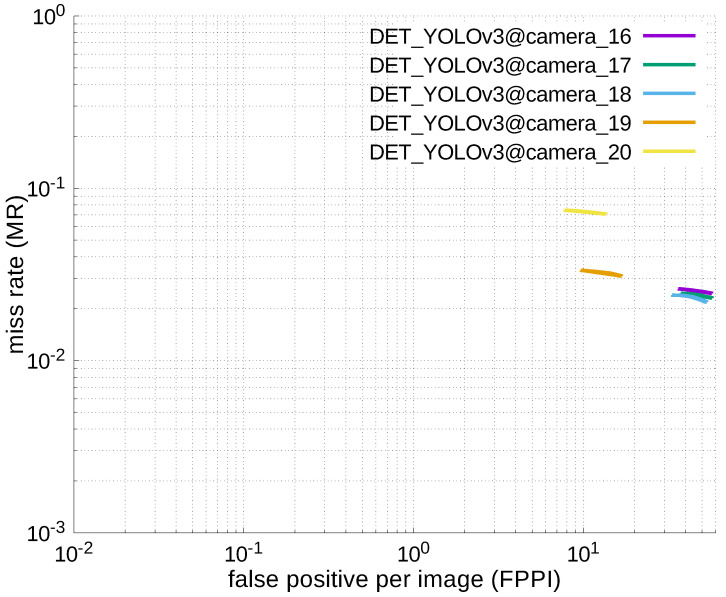
DET curves @ Cameras_16–20, YOLOv3.

Using the results in the tables, a detection error tradeoff (DET) curve, representing false positive rates versus miss rates, was plotted to show the detection performance at several classification thresholds to evaluate miss rates more densely. The DET graphs in Figure 15, Figure 16, Figure 17, Figure 18 and Figure 19 and Figure 20, Figure 21, Figure 22, Figure 23 and Figure 24 show that the miss rates of our scheme using informed-filters was much better than YOLOv3, a state-of-the-art scheme.

#### 4.2.4. Discussion

As shown in Table 6, Table 7, Table 8, Table 9 and Table 10, these results were caused by the weak point of YOLO: it cannot well-detect small targets. Whereas YOLOv3 adopts feature-pyramid networks [20] to improve the detection performance for small targets, the performance of YOLOv3 was still insufficient to detect such targets. This problem can be resolved if the grid becomes finer. However, it increases required computational power.

Moreover, most elements in the tables were “nan”, indicating that FPPI by YOLOv3 were quite large for all cameras, because the grid-based detection scheme did not work well when the size of detection target was small compared to the size of an input image. However, informed-filters showed good accuracy with very low false positives. The experimental results show that a carefully trained detector, without deep learning, could achieve good detection accuracy.

Notwithstanding the advantage of detection accuracy, informed-filters had another merit: low required computational power. YOLOv3 can achieve fast computation, but requires massive GPUs that cannot be implemented in a real-time system mounted on UAVs. Therefore, the detection scheme based on informed-filters is more appropriate for our target application.

### 4.3. Detection Accuracy by Informed-Filters When Detectors Are Constructed Using Only Samples Extracted from Particular Views

A detector was constructed using only training samples extracted from all views. However, the appearance of targets changes drastically when a view changes. Thus, we constructed detectors specialized for only some viewpoints, and we evaluated the detection accuracy changes compared to the detector constructed, using all views.

#### 4.3.1. Experimental Setup

Before the evaluation, all views were divided into five groups: Camera_00, Cameras_01–05, Cameras_06–10, Cameras_11–15, Cameras_16–20, and detectors were trained in each. In this experiment, the same dataset and evaluation criteria as the previous experiment were adopted.

#### 4.3.2. Experimental Results

**Figure 25:**
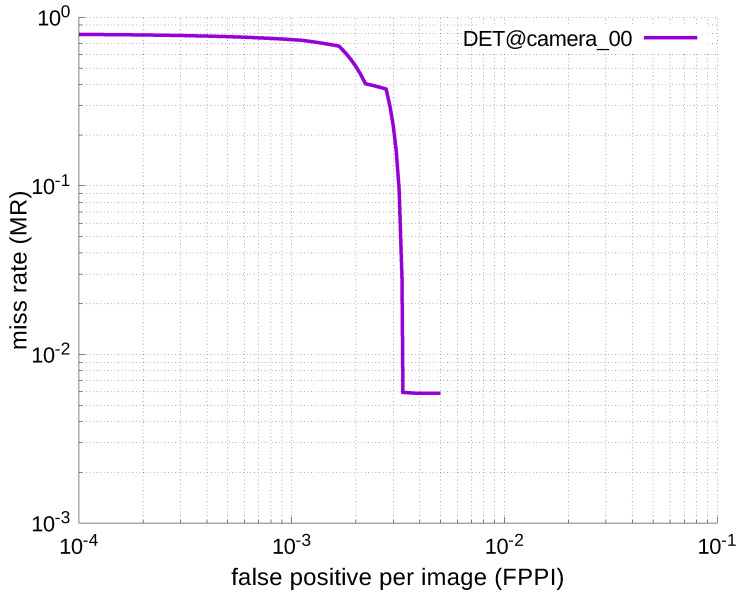
DET curves @ Camera_00, informed-filters, some viewpoints.

**Figure 26:**
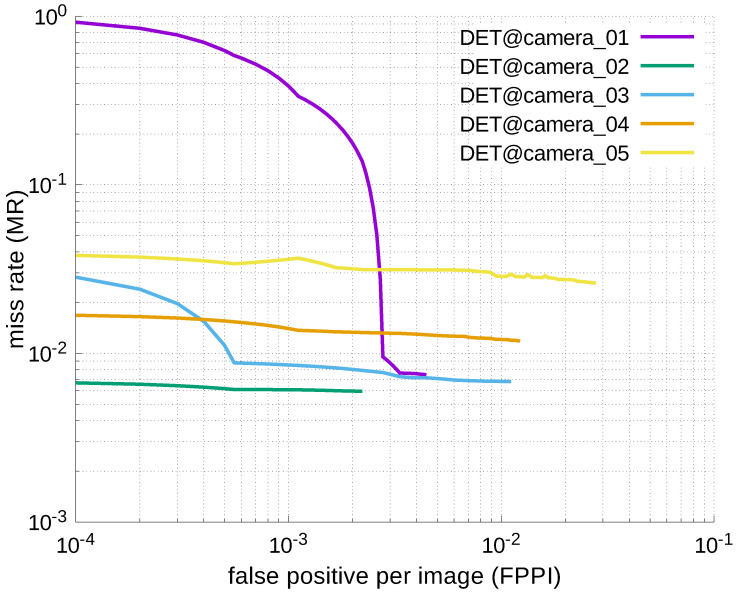
DET curves @ Cameras_01–05, informed-filters, some viewpoints.

**Figure 27:**
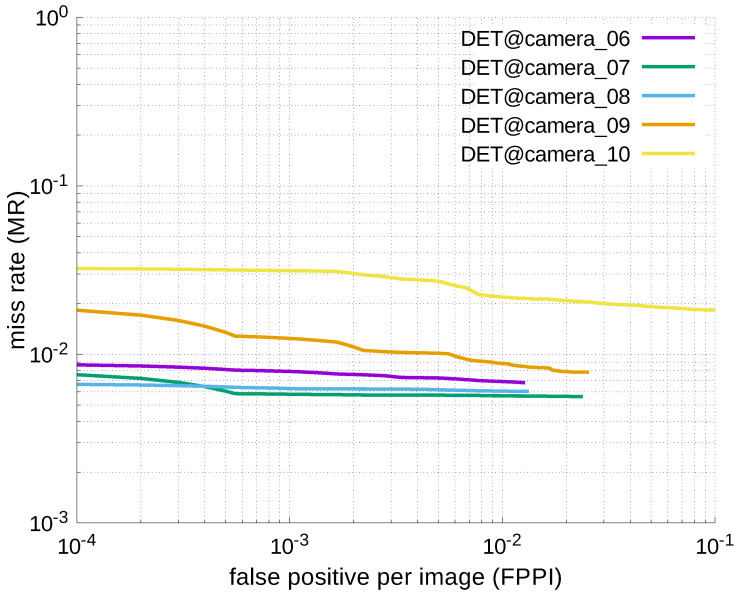
DET curves @ Cameras_06–10, informed-filters, some viewpoints.

**Figure 28:**
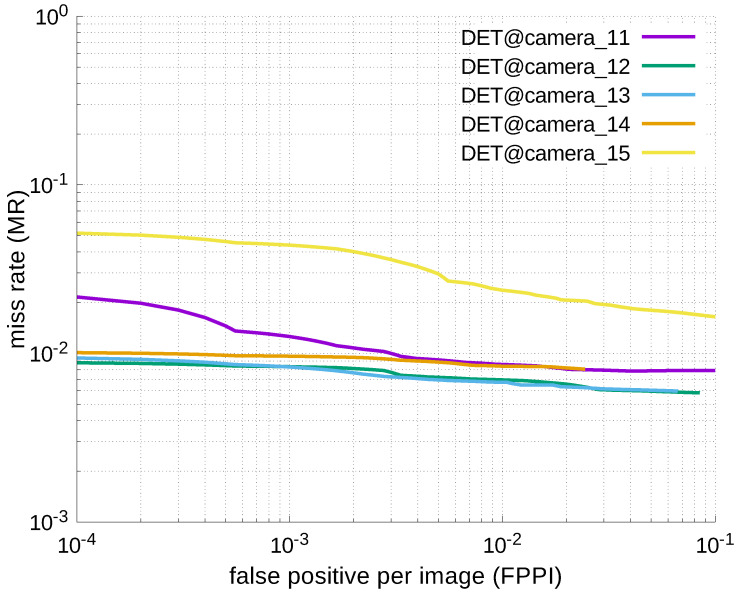
DET curves @ Cameras_11–15, informed-filters, some viewpoints.

**Figure 29:**
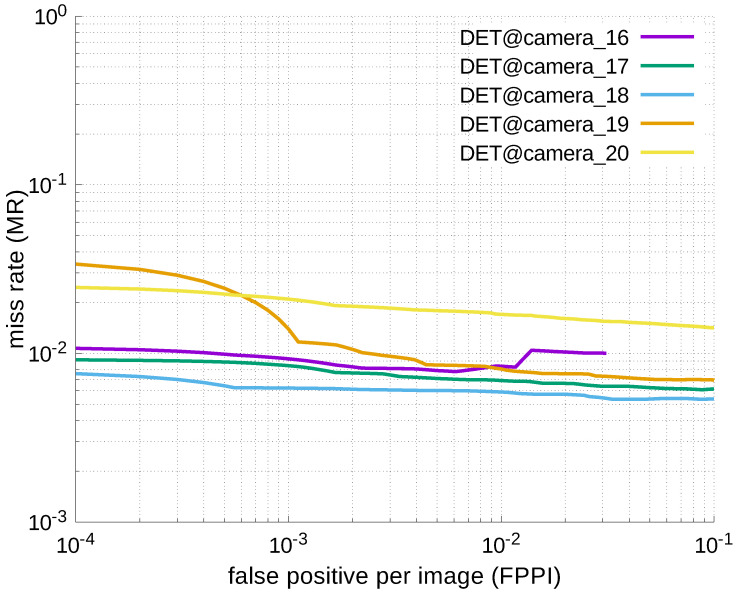
DET curves @ Cameras_16–20, informed-filters, some viewpoints.

Table 11, Table 12, Table 13, Table 14 and Table 15 show four miss rates at the specified FPPIs for views explained in the previous subsection. Figure 25, Figure 26, Figure 27, Figure 28 and Figure 29 show DET curves corresponding to constructed detectors. In the tables, a similar trend with the results of using all views was observed. However, accuracy was improved for some views, whereas miss rates were exceedingly bad in the previous experiment: Camera_00, Camera_01, and viewpoints with a low height.

This improvement of detection accuracy, when detectors were trained using only samples obtained from appropriate views became remarkable at lower FPPI. Sudden changes of detection accuracy caused by detectors were reduced (for example, Camera_14).

#### 4.3.3. Discussion

These results indicate that multiple detectors at different views should be adopted to achieve better detection instead of only a single detector, which uses all samples extracted from several views. Regarding cases where the miss rate was originally low, there were very slight changes.

As a result, more stable detection was achieved using the whole multiple detector. However, dramatic accuracy improvement cannot be expected.

## 5. Conclusions

This paper evaluated the detection accuracy of players from many viewpoints to find the best UAV locations for capturing aerial images. For the evaluation, several kinds of 2D images with annotations about player locations were generated from the 3D virtual soccer field using orientations and locations of a UAV camera. To train a strong classifier, a large template pool was created from several template pools, one of which was generated from viewpoints whose shooting orientation was the same.

Experimental results using the generated 2D images show that the detection accuracy becomes best when a camera is located at a viewpoint slightly distant from just above the center of the field. Additionally, it is possible to perform more stable detection, if viewpoints are divided into groups appropriately, and if multiple detectors are constructed for each group.

## Figures and Tables

**Table 1:** Accuracy table @ Camera_00, informed-filters.

	MR@Camera_00
FPPI = 1.0	0.000000
FPPI = 0.1	0.000000
FPPI = 0.01	0.006490
FPPI = 0.001	0.812667

**Table 2:** Accuracy table @ Cameras_01–05, informed-filters.

	MR@Camera_01	MR@Camera_02	MR@Camera_03	MR@Camera_04	MR@Camera_05
FPPI = 1.0	0.000000	0.000000	0.000000	0.000000	0.000000
FPPI = 0.1	0.000000	0.000000	0.000000	0.000000	0.000000
FPPI = 0.01	0.006414	0.000000	0.008468	0.015025	0.031540
FPPI = 0.001	0.558980	0.007738	0.014591	0.033021	0.037637

**Table 3:** Accuracy table @ Cameras_06–10, informed-filters.

	MR@Camera_06	MR@Camera_07	MR@Camera_08	MR@Camera_09	MR@Camera_10
FPPI = 1.0	0.000000	0.005433	0.000000	0.000000	0.000000
FPPI = 0.1	0.000000	0.005479	0.000000	0.000000	0.021780
FPPI = 0.01	0.000000	0.005484	0.006148	0.009432	0.027652
FPPI = 0.001	0.005678	0.005683	0.007467	0.012366	0.036157

**Table 4:** Accuracy table @ Cameras_11–15, informed-filters.

	MR@Camera_11	MR@Camera_12	MR@Camera_13	MR@Camera_14	MR@Camera_15
FPPI = 1.0	0.000000	0.000000	0.000000	0.000000	0.000000
FPPI = 0.1	0.000000	0.000000	0.000000	0.006938	0.017012
FPPI = 0.01	0.006399	0.005524	0.006243	0.012166	0.024646
FPPI = 0.001	0.007465	0.006269	0.012894	0.059582	0.039167

**Table 5:** Accuracy table @ Cameras_16–20, informed-filters.

	MR@Camera_16	MR@Camera_17	MR@Camera_18	MR@Camera_19	MR@Camera_20
FPPI = 1.0	0.000000	0.000000	0.000000	0.006879	0.000000
FPPI = 0.1	0.000000	0.000000	0.000000	0.006982	0.015134
FPPI = 0.01	0.000000	0.000000	0.005956	0.008233	0.020135
FPPI = 0.001	0.006808	0.005627	0.009709	0.043803	0.039258

**Table 6:** Accuracy table @ Camera_00, YOLOv3.

	MR@Camera_00
FPPI = 1.0	0.031323
FPPI = 0.1	nan
FPPI = 0.01	nan
FPPI = 0.001	nan

**Table 7:** Accuracy table @ Cameras_01–05, YOLOv3.

	MR@Camera_01	MR@Camera_02	MR@Camera_03	MR@Camera_04	MR@Camera_05
FPPI = 1.0	nan	nan	nan	nan	nan
FPPI = 0.1	nan	nan	nan	nan	nan
FPPI = 0.01	nan	nan	nan	nan	nan
FPPI = 0.001	nan	nan	nan	nan	nan

**Table 8:** Accuracy table @ Cameras_06–10, YOLOv3.

	MR@Camera_06	MR@Camera_07	MR@Camera_08	MR@Camera_09	MR@Camera_10
FPPI = 1.0	nan	nan	nan	nan	nan
FPPI = 0.1	nan	nan	nan	nan	nan
FPPI = 0.01	nan	nan	nan	nan	nan
FPPI = 0.001	nan	nan	nan	nan	nan

**Table 9:** Accuracy table @ Cameras_11–15, YOLOv3.

	MR@Camera_11	MR@Camera_12	MR@Camera_13	MR@Camera_14	MR@Camera_15
FPPI = 1.0	nan	nan	nan	nan	nan
FPPI = 0.1	nan	nan	nan	nan	nan
FPPI = 0.01	nan	nan	nan	nan	nan
FPPI = 0.001	nan	nan	nan	nan	nan

**Table 10:** Accuracy table @ Cameras_16–20, YOLOv3.

	MR@Camera_16	MR@Camera_17	MR@Camera_18	MR@Camera_19	MR@Camera_20
FPPI = 1.0	nan	nan	nan	nan	nan
FPPI = 0.1	nan	nan	nan	nan	nan
FPPI = 0.01	nan	nan	nan	nan	nan
FPPI = 0.001	nan	nan	nan	nan	nan

**Table 11:** Accuracy table @ Camera_00, informed-filters, some viewpoints.

	MR@Camera_00
FPPI = 1.0	0.000000
FPPI = 0.1	0.000000
FPPI = 0.01	0.000000
FPPI = 0.001	0.737169

**Table 12:** Accuracy table @ Cameras_01–05, informed-filters, some viewpoints.

	MR@Camera_01	MR@Camera_02	MR@Camera_03	MR@Camera_04	MR@Camera_05
FPPI = 1.0	0.000000	0.000000	0.000000	0.000000	0.000000
FPPI = 0.1	0.000000	0.000000	0.000000	0.000000	0.000000
FPPI = 0.01	0.000000	0.000000	0.006818	0.012108	0.028662
FPPI = 0.001	0.385389	0.006091	0.008546	0.014050	0.036182

**Table 13:** Accuracy table @ Cameras_06–10, informed-filters, some viewpoints.

	MR@Camera_06	MR@Camera_07	MR@Camera_08	MR@Camera_09	MR@Camera_10
FPPI = 1.0	0.000000	0.000000	0.000000	0.000000	0.000000
FPPI = 0.1	0.000000	0.000000	0.000000	0.000000	0.018324
FPPI = 0.01	0.006900	0.005680	0.006053	0.008847	0.021843
FPPI = 0.001	0.007918	0.005801	0.006270	0.012433	0.031384

**Table 14:** Accuracy table @ Cameras_11–15, informed-filters, some viewpoints.

	MR@Camera_11	MR@Camera_12	MR@Camera_13	MR@Camera_14	MR@Camera_15
FPPI = 1.0	0.000000	0.000000	0.000000	0.000000	0.000000
FPPI = 0.1	0.007914	0.000000	0.000000	0.000000	0.016500
FPPI = 0.01	0.008600	0.006971	0.006742	0.008397	0.023712
FPPI = 0.001	0.012590	0.008344	0.008309	0.009631	0.043875

**Table 15:** Accuracy table @ Cameras_16–20, informed-filters, some viewpoints.

	MR@Camera_16	MR@Camera_17	MR@Camera_18	MR@Camera_19	MR@Camera_20
FPPI = 1.0	0.000000	0.000000	0.000000	0.000000	0.000000
FPPI = 0.1	0.000000	0.006160	0.005368	0.006952	0.014206
FPPI = 0.01	0.008388	0.006904	0.005902	0.008081	0.017039
FPPI = 0.001	0.009280	0.008454	0.006228	0.013950	0.020981
